# Multi-predictor modeling for predicting early Parkinson’s disease and non-motor symptoms progression

**DOI:** 10.3389/fnagi.2022.977985

**Published:** 2022-08-26

**Authors:** Kaixin Dou, Jiangnan Ma, Xue Zhang, Wanda Shi, Mingzhu Tao, Anmu Xie

**Affiliations:** Department of Neurology, Affiliated Hospital of Qingdao University, Qingdao, China

**Keywords:** Parkinson’s disease, predictive model, diagnosis, non-motor symptoms, progression

## Abstract

**Background:**

Identifying individuals with high-risk Parkinson’s disease (PD) at earlier stages is an urgent priority to delay disease onset and progression. In the present study, we aimed to develop and validate clinical risk models using non-motor predictors to distinguish between early PD and healthy individuals. In addition, we constructed prognostic models for predicting the progression of non-motor symptoms [cognitive impairment, Rapid-eye-movement sleep Behavior Disorder (RBD), and depression] in *de novo* PD patients at 5 years of follow-up.

**Methods:**

We retrieved the data from the Parkinson’s Progression Markers Initiative (PPMI) database. After a backward variable selection approach to identify predictors, logistic regression analyses were applied for diagnosis model construction, and cox proportional-hazards models were used to predict non-motor symptom progression. The predictive models were internally validated by correcting measures of predictive performance for “optimism” or overfitting with the bootstrap resampling approach.

**Results:**

For constructing diagnostic models, the final model reached a high accuracy with an area under the curve (AUC) of 0.93 (95% CI: 0.91–0.96), which included eight variables (age, gender, family history, University of Pennsylvania Smell Inventory Test score, Montreal Cognitive Assessment score, RBD Screening Questionnaire score, levels of cerebrospinal fluid α-synuclein, and *SNCA* rs356181 polymorphism). For the construction of prognostic models, our results showed that the AUC of the three prognostic models improved slightly with increasing follow-up time. The overall AUCs fluctuated around 0.70. The model validation established good discrimination and calibration for predicting PD onset and progression of non-motor symptoms.

**Conclusion:**

The findings of our study facilitate predicting the individual risk at an early stage based on the predictors derived from these models. These predictive models provide relatively reliable information to prevent PD onset and progression. However, future validation analysis is still needed to clarify these findings and provide more insight into the predictive models over more extended periods of disease progression in more diverse samples.

## Introduction

Parkinson’s disease (PD, OMIM 168600), the most common motoric neurodegenerative disease, affects approximately 1–2% of people older than 60 years (Wirdefeldt et al., 2011; Ascherio and Schwarzschild, 2016). When a classical feature of bradykinesia is present and combined with other features such as rigidity, tremor, and postural instability, a clear clinical diagnosis of PD can be made (Postuma et al., 2015). Although PD is categorized as a movement disorder, non-motor symptoms (for instance, olfactory disorders, constipation, and sleep disorders) indeed frequently occur in the early stage of the disease, and may even precede the emergence of motor dysfunction (Postuma et al., 2012; Schapira et al., 2017). Non-motor symptoms are almost inevitably linked to the later development of diseases and a lower quality of life (Schrag et al., 2000; Santos García et al., 2021). With the increasing understanding of PD as a multi-system heterogeneous disorder, the modern scientific diagnosis should include the assessment and management of non-motor symptoms (Chaudhuri et al., 2006). There is inconsistency in the manifestation of these symptoms and their rate of progression in PD patients, which presents a challenge for researchers developing drugs to modify the disease process (Olanow et al., 2009; Smedinga et al., 2021). Therefore, evaluating non-motor symptoms, especially in the prodromal and early stages of the disease, can help determine whether individuals are at risk for developing PD or later complications.

In the past decades, researchers have made many efforts to identify patients with PD during the prodromal and early periods of the disease, such as the Parkinson Progression Marker Initiative (2011). A few recent diagnostic and prognostic models have been generated that are devoted to predicting different aspects of PD symptomatology specifically (Ma et al., 2020; Ren et al., 2021). Most previous diagnostic models focused on various clinical motor scores as predictors (Nalls et al., 2015; Searles Nielsen et al., 2017; Schrag et al., 2019). However, given the heterogeneous nature of motor symptoms, the predictive accuracy of many existing risk models is only moderate or even low (Fereshtehnejad et al., 2015; Searles Nielsen et al., 2017). Considering the potential value of non-motor symptoms for early identification, developing the predictive models utilizing non-motor predictors may identify high-risk PD groups. Olfactory dysfunction and Rapid-eye-movement sleep Behavior Disorder (RBD) are the most common non-motor symptoms. The findings from a prospective study suggested that 10% of individuals with olfactory dysfunction developed PD during a 2-year follow-up, and thus idiopathic hyposmia can be as a preclinical sign of PD (Ponsen et al., 2004). Many candidate biomarkers have been tested in different study cohorts, such as cerebrospinal fluid (CSF) α-synuclein (α-syn), amyloid-β_42_ (Aβ_42_), and total tau (t-tau) (Parnetti et al., 2013). Recently, CSF or serum neurofilament light chain (NFL) as one promising candidate biomarker has added diagnostic value to biomarker panels (Oosterveld et al., 2020).

From observational and longitudinal studies, several non-motor symptoms affecting cognition, sleep, and psychosis appeared to be correlated with the later progression of PD (Schrag et al., 2015; Galtier et al., 2019). Dementia is one of the most common non-motor symptoms of PD, with a prevalence of about 30% (Aarsland et al., 2005). The cumulative incidence of dementia steadily increases with age and duration of PD, increasing to 80% by age 90 (Buter et al., 2008). RBD is another common non-motor symptom reported in all stages of PD, with a combined prevalence of 42% (Zhang et al., 2017). In total, 10–45% of PD patients will become depressed as the disease progresses (Lemke, 2008). The progression of non-motor symptoms has dominant-negative effects on health-related quality of life, thus identifying prognostic factors of non-motor symptoms is vital for minimizing impairments and proposing the personalization of PD management (Gómez-Esteban et al., 2011; Duncan et al., 2014). Yet lack of longitudinal assessments in a few previous studies, there was no fuller insight into the potential role of developing comprehensive multivariable prognostic models.

Our goals in the present study were to: (i) establish and validate the most explanatory model on a baseline dataset to diagnose early PD groups with non-motor clinical characteristics, biomarkers and genetic information; (ii) construct prognostic models for predicting the progression of non-motor symptoms in *de novo* PD patients, for instance, cognitive impairment, RBD and depression at 5 years of follow-up; and (iii) identify the predictors in order to better predict individual prognosis and guide the prevention.

## Materials and methods

### Parkinson’s Progression Markers Initiative database and participants

In the present study, we retrieved the data from the PPMI database. The PPMI is a global, multicenter, prospective research with the design goal of investigating and verifying biomarkers that may slow the disease progression. As described previously, the study investigated drug-naive, *de novo* PD patients and age- and gender-matched healthy controls (HCs) between June 2010 and May 2013 (2011). The participants with PD were recruited if they met the following requirements: (1) age older than 30 years; (2) existence of two symptoms as below: bradykinesia, rigidity, resting tremor, or asymmetric resting tremor, or asymmetric bradykinesia; (3) diagnosis recently made within the last 2 years; (4) PD drug naivety; and (5) dopamine transporter deficit in the putamen on the DaTscan by central reading. HCs were required to meet the following criteria: no significant neurological dysfunction, no first-degree relatives with PD and Montreal Cognitive Assessment (MoCA) score above 26. In this article, the baseline dataset used for diagnostic models and the 5-year follow-up dataset used for constructing progression models were downloaded on 5 March 2022. More detailed information could be sought at http://ppmi-info.org/.

### Standard protocol approvals, registrations, and patient consent

The study was approved in all participating sites, respectively, by each local ethical standards committee on human experimentation as described (Lee et al., 2019). Written informed consent for research was obtained from all study participants. The PPMI study is registered with clinicaltrials.gov_(identifier: NCT01141023).

### Cerebrospinal fluid and blood biomarker assessments

Biomarker analyses have been previously described and based on the PPMI biologics manual (Marek et al., 2018). CSF was collected by standardized lumbar puncture procedures. Measurements of Aβ_42_ and t-tau were analyzed by using the xMAP-Luminex platform with INNOBIA AlzBio3 immunoassay kit–based reagents (Fujirebio-Innogenetics, Ghent, Belgium) at Penn (Kang et al., 2013). Additionally, CSF total α-syn levels were measured by BioLegend (San Diego, CA, United States) by means of a commercially accessible and previously described sandwich immunoassay. Serum NFL was quantified by the Simoa Human NF-light Advantage Kit (Quanterix, Lexington, MA, United States) using the Single Molecule Array technology in a fully automated SIMOA HD-1 analyzer. Biochemical analyses of uric acid have been carried out in Covance laboratories in a uniform fashion, as per the study protocol.

### Genetic assessments

We analyzed genetic data for *MAPT*, and single-nucleotide polymorphisms related to *SNCA* (rs3910105 and rs356181), provided from the PPMI Genetics Core. SNPs of *SNCA* and *MAPT* genes were determined using Illumina NeuroX array on whole-blood extracted DNA per manufacturer’s protocol (Illumina Inc., San Diego, CA, United States) (Nalls et al., 2016).

### Predictor variables

Predictive variables included demographic data (age, gender, years of education, family history, age at onset, and ethnicity), disease duration, risk gene (*SNCA*_rs356181, *SNCA*_rs3910105, and *MAPT* status) and measures of non-motor function. For non-motor symptoms evaluation, sleep quality and disturbances of patients were measured by the Epworth Sleepiness Scale score (ESS) and RBD Screening Questionnaire score (RBDSQ). MoCA was the most common screening instrument for cognitive function. The University of Pennsylvania Smell Inventory Test (UPSIT) score was applied to assess olfactory function. The Questionnaire for Impulsive-Compulsive Disorders in Parkinson’s Disease (QUIP) score was a rating scale designed to evaluate impulse control disorders. The Geriatric Depression Scale (GDS) score was applied to assess depression. The global motor impairment was assessed using total score and section III of the Movement Disorder Society-Sponsored Revision of the Unified Parkinson’s Disease Rating Scale (MDS-UPDRS). In addition, we assessed whether biomarkers including CSF Aβ_42_, t-tau and CSF α-syn, serum uric acid, and NFL had impacts on the risk models in this study.

### Longitudinal assessments

All the above predictive variables are remeasured annually. We constructed 5-year longitudinal models to evaluate the progression of non-motor symptoms (cognitive impairment, RBD, and depression) in early PD. Patients with less than 1-year follow-up were excluded from the longitudinal analyses. For constructing three prognostic models, we excluded PD patients who had corresponding outcome status at baseline. In the present study, defining the presence of non-motor symptoms was determined by several neuropsychological assessments. Cognitive status was defined based on the MDS task force level II guideline criteria: PD with normal cognition (PD-NC) if MoCA > 26, PD with mild cognitive impairment (PD-MCI) if MoCA between 23 and 26, and PD with dementia (PD-D) if MoCA < 23 (Litvan et al., 2012). During the longitudinal follow-up, PD-NC patients who progressed to PD-MCI or PD-D were classified as “cognitive impairment progression.” The RBDSQ consists of 10 questions with 13 items overall, and RBD positive was defined if RBDSQ was above 6-points. RBD-negative patients who progressed to RBD positive were considered to have “RBD progression.” The GDS is a self-report questionnaire for rating depressive symptoms, with a score of ≥5 indicating clinically significant depression in PD. Progression from no depressive symptoms at baseline to depressive symptoms was considered “depressive progression.”

In the PPMI cohort, individuals were evaluated at baseline and followed for 5 years, during which some subjects started dopamine replacement therapy (DRT) such as levodopa, dopamine agonist, and others. The prescribed dose of DRT at the 5th year follow-up was expressed as levodopa equivalent daily dose (LEDD) in milligrams (mg). Therefore, we added LEDD as a candidate predictor in the prognostic model to examine the effect of DRT on the progression of non-motor symptoms.

### Statistical analyses

All statistical analyses were performed by R software (version 4.1.3). Baseline characteristics were presented as mean ± standard deviation (SD) or number (percentages, %), as appropriate. Differences cross groups were evaluated by the Mann–Whitney–Wilcoxon test for continuous variables and the Chi-square test for categorical variables. Missing values were excluded from the analysis. For constructing diagnostic models, all candidate risk factors were entered into the logistic regression analysis and the assumption of proportional hazards was confirmed. Additionally, we used multivariate cox proportional-hazards models to predict non-motor symptom progression. A backward variable selection approach with a cut-off value at the *P* < 0.05 was used to identify the set of independent predictors. Final variables were tested by the Spearman rank correlation analysis to ensure that Spearman’s correlation coefficients of no more than 0.5. Receiver operating characteristics analysis (ROC), the area under the ROC curve (AUC), sensitivity and specificity at each optimal cut-off value were applied to assess the model performance. The statistical analyses of ROC curves were carried out using the “pROC” packages in R. Model calibration was evaluated by generating a smooth curve in the calibration plot between the observed and predicted outcomes. The calibration plot would equal 1 (optimization criteria) if the observed and predicted probabilities represent perfect agreement. The predictive models were internally validated by correcting measures of predictive performance for “optimism” or overfitting with bootstrap resampling approach (with 1,000 replicas) in the *rms* packages in R.

## Results

### Demographic and clinical characteristics of included participants

**FIGURE 1:**
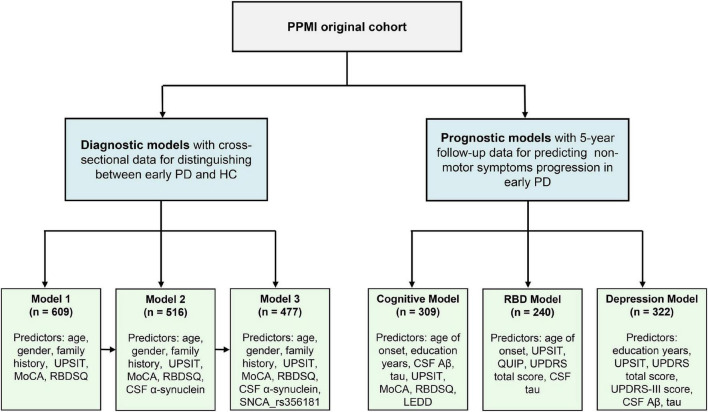
Flowchart of data analysis. PPMI, Parkinson’s Progression Markers Initiative; PD, Parkinson’s disease; MoCA, Montreal Cognitive Assessment; UPSIT, University of Pennsylvania Smell Inventory Test; RBM, Rapid-eye-movement sleep Behavior Disorder Screening Questionnaire; MDS-UPDRS, Movement Disorder Society-Sponsored Revision of the Unified Parkinson’s Disease Rating Scale; CSF, cerebrospinal fluid; Aβ, amyloid-β.

Figure 1 presents a flowchart outlining the main analysis in this study. In the risk model for distinguishing between early PD and healthy normal, a total of 194 HC individuals and 415 *de novo* PD patients were included. The baseline characteristics of participants are shown in Table 1. For clinical characteristics, no significant differences in age (*P* = 0.65), gender (*P* = 0.66), education level (*P* = 0.08), ethnicity (*P* = 0.61), ESS score (*P* = 0.28), and QUIP score (*P* = 0.62) were found between the two diagnostic groups. While the family history of PD, Hoehn and Yahr, MoCA score, UPSIT score, GDS score, RBDSQ score, and UPDRS score differed between groups (*P* < 0.05). For CSF and blood biomarkers, CSF α-syn (*P* = 0.01), Aβ_42_ (*P* = 0.05), and t-tau (*P* = 0.003) were lower in the PD group compared to control group, but serum NFL levels were higher in the *de novo* PD group than control. For genetic characteristics, only *SNCA* rs356181 status differed between two diagnostic groups (*P* = 0.005). Using longitudinal data to predict non-motor symptoms progression in *de novo* PD patients, three prognostic models were performed separately to estimate the risk of cognition, RBD, and depression. The three models included 309, 240, and 322 *de novo* PD patients, respectively. The baseline characteristics of three prognostic models are summarized in Supplementary Table 1.

**TABLE 1:** Baseline demographic and disease characteristics of included participants.

Characteristics	PD subjects (*n* = 415)	HC subjects (*n* = 194)	*P-value*
**Demographic and clinical characteristics**			
Age (mean, SD)	61.7 (9.7)	60.9 (11.2)	0.65
Gender (male/female)	272/143	124/70	0.66
Education years (mean, SD)	15.6 (3.0)	16.1 (2.9)	0.08
Family history of PD (any family with PD/no family with PD)	102/313	10/184	<0.0001
Ethnicity (Hispanic or Latino/not Hispanic or Latino)	9/406	3/191	0.61
Age of PD onset	59.5 (10.0)	NA	NA
Hoehn and Yahr			<0.0001
Stage 0	0	192	
Stage 1	182	2	
Stage 2	231	0	
Stage 3–5	2	0	
MoCA score (mean, SD)	27.1 (2.3)	28.2 (1.1)	<0.0001
UPSIT score (mean, SD)	22.3 (8.3)	34.0 (4.9)	<0.0001
RBDSQ score (no RBD/RBD)	258/157	156/38	<0.0001
GDS score (not depressed/depressed)	356/59	194/0	0.01
ESS score (not sleepy/sleepy)	350/65	156/38	0.28
QUIP score (mean, SD)	0.3 (0.6)	0.3 (0.7)	0.62
MDS-UPDRS Part I score (mean, SD)	5.6 (4.1)	1.2 (2.2)	<0.0001
MDS-UPDRS Part II score (mean, SD)	5.9 (4.2)	0.5 (1.0)	<0.0001
MDS-UPDRS Part III score (mean, SD)	20.8 (8.8)	2.9 (3.0)	<0.0001
MDS-UPDRS total score (mean, SD)	32.2 (13.1)	4.6 (4.5)	<0.0001
**CSF and blood markers**			
α-Synuclein (pg/ml, mean, SD)	1,550.7 (687.2)	1,703.8 (731.8)	0.01
Aβ_42_ (pg/ml, mean, SD)	931.8 (420.5)	1,030.8 (504.0)	0.05
Total tau (pg/ml, mean, SD)	171.1 (59.0)	193.8 (80.1)	0.003
Urate (pg/ml, mean, SD)	313.8 (75.6)	322.7 (78.4)	0.18
NFL (pg/ml, mean, SD)	13.1 (7.2)	11.9 (6.7)	0.03
**Genetic characteristics**			
*SNCA*_rs356181			0.005
C/C	114	32	
C/T	183	95	
T/T	86	51	
Missing	32	16	
*SNCA*_rs356105			0.09
C/C	63	44	
C/T	197	82	
T/T	123	52	
Missing	32	16	
*MAPT*			0.77
H1/H1	240	114	
H1/H2	126	56	
H2/H2	17	8	
Missing	32	16	

PD, Parkinson’s disease; HC, healthy control; SD, standard deviation; MoCA, Montreal Cognitive Assessment; UPSIT, University of Pennsylvania Smell Inventory Test; RBDSQ, Rapid-eye-movement sleep Behavior Disorder Screening Questionnaire; ESS, Epworth Sleeping Scale; GDS, Geriatric Depression Scale; MDS-UPDRS, Movement Disorder Society-Sponsored Revision of the Unified Parkinson’s Disease Rating Scale; NFL, neurofilament light; CSF, cerebrospinal fluid; Aβ_42_, amyloid-β_42_.

### Predictive modeling for distinguishing between early Parkinson’s disease and healthy normal

**FIGURE 2:**
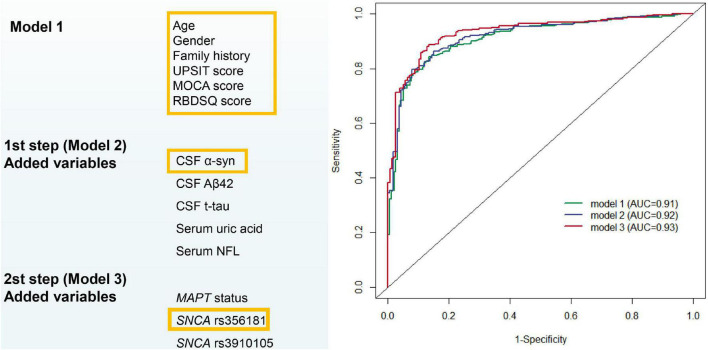
Predictive modeling for distinguishing between early Parkinson’s disease and healthy normal. AUC, area under receiver operating characteristic curves.

The first model was constructed with demographics, neuropsychological tests and health variables which can be easily available from primary clinical assessments. After stepwise logistic regression, we retained six PD risk factors in Model 1: age, gender, family history of PD, total UPSIT score, the MoCA score and the RBDSQ score (see Supplementary Table 2). None of these risk factors were used as part of the PD diagnosis criteria. The model has acceptable accuracy for predicting whether subsets of healthy individuals with abnormal baseline linical characteristics will develop *de novo* PD; the AUC was 0.91 (95% CI: 0.89–0.94, sensitivity 86.6% and specificity 84.4%, Figure 2).

Cerebrospinal fluid biomarkers (α-syn, Aβ_42_, and t-tau) and blood biomarkers (serum uric acid and NFL) were included as possible variables besides the easily available variables used in the construction of Model 2. After variable selection by backward stepwise logistic regression, Model 2 included age, gender, family history of PD, total UPSIT score, the MoCA score, RBDSQ score, and CSF α-syn (Supplementary Table 2). The AUC was improved to 0.92 (95% CI: 0.89–0.94, sensitivity 79.7% and specificity 92.2%, Figure 2) with the inclusion of new variables.

**FIGURE 3:**
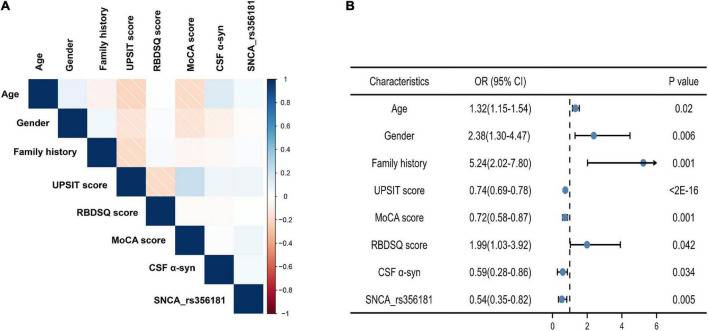
The eight predictors in the final diagnostic model. **(A)** Correlation heatmap between the eight variables; **(B)** multivariate analysis of eight variables in the diagnostic model. MoCA, Montreal Cognitive Assessment; UPSIT, University of Pennsylvania Smell Inventory Test; RBM, Rapid-eye-movement sleep Behavior Disorder Screening Questionnaire; CSF α-syn, cerebrospinal fluid α-synuclein.

Model 3 evaluated the genetic status (*MAPT* status, *SNCA* rs356181, and *SNCA* rs3910105) of PD adjusted for the covariates included in Model 2 to acquire more accurate calculation in predicting risk individuals. Age, gender, family history of PD, total UPSIT score, the MoCA score, RBDSQ score, CSF α-syn, and *SNCA* rs356181 polymorphism were selected as final variables in Model 3, and Model 3 as the final diagnostic model predicted early PD in this study. In this analysis, the ROC curves demonstrated an AUC of 0.93 (95% CI: 0.91–0.96, Figure 2) with a sensitivity of 88.1% and a specificity of 87.3%. Figure 3A described the heatmap of the Spearman correlation coefficients between the final variables, and there was no strong correlation between the final eight included variables (Supplementary Table 3). All variables were superior predictive indicators in the multifactorial analyses (*P* < 0.05, Figure 3B).

### Internal validation and calibration of the diagnostic model

Finally, Model 3 was selected as the prediction model for distinguishing early PD from healthy normal, and we conducted model-fitting analysis and internal validation based on Model 3. This model showed calibration (calibration slope, 1; Brier score, 0.10; Hosmer–Lemeshow χ^2^ = 15.17; *P* = 0.06, Supplementary Figure 1). Internal validation showed minimal mean optimism of 0.008 with bootstrap optimism corrected AUC of 0.92 based on 1000 resamplings.

### Prognostic modeling for predicting non-motor symptoms progression in *de novo* Parkinson’s disease patients

Candidate variables used in the construction of cognitive decline, depression and RBD model included age, gender, education years, ethnicity, family history, age at symptom onset, disease duration, UPSIT score, GDS score, QUIP score, MoCA score, CSF Aβ, α-syn, t-tau, MDS-UPDRS total score, MDS-UPDRS Part III score, ESS score, RBDSQ score, and level of LEDD.

**FIGURE 4:**
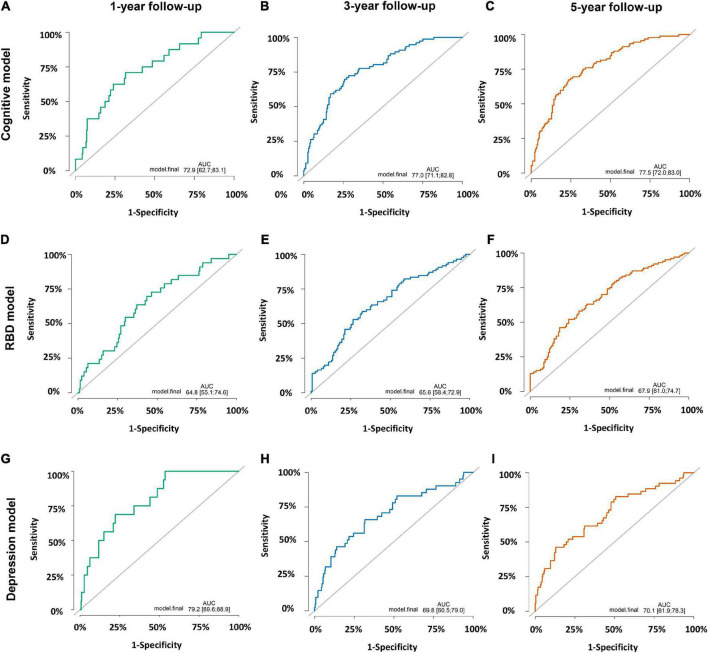
Prediction accuracies of three prognostic models. **(A)** Cognitive decline model within 1 year with area under receiver operating characteristic curves (AUC) of 0.73 (95% CI: 0.63–0.83). **(B)** Cognitive decline model within 3 years with AUC of 0.77 (95% CI: 0.71–0.83). **(C)** Cognitive decline model within 5 years with AUC of 0.78 (95% CI: 0.72–0.83). **(D)** Rapid-eye-movement sleep Behavior Disorder (RBD) prognostic model within 1 year with AUC of 0.65 (95% CI: 0.55–0.75). **(E)** RBD prognostic model within 3 years with AUC of 0.66 (95% CI: 0.58–0.73). **(F)** RBD prognostic model within 5 years with AUC of 0.68 (95% CI: 0.61–0.75). **(G)** Depression prognostic model within 1 year with AUC of 0.79 (95% CI: 0.70–0.89). **(H)** Depression prognostic model within 3 years with AUC of 0.70 (95% CI: 0.61–0.79). **(I)** Depression prognostic model within 5 years with AUC of 0.70 (95% CI: 0.62–0.78).

Of the 309 early PD patients with cognitive normal at baseline were included in the cognitive decline model with eight variables (age at symptom onset, education years, MoCA score, UPSIT score, RBDSQ score, LEDD and levels of CSF Aβ_42_, and t-tau, Supplementary Table 4). As shown in Figure 4, the model predicted the incident of cognitive decline within 1, 3, and 5 years with moderate accuracy (AUC of 0.73, 0.77, and 0.78, respectively, Figures 4A–C).

A subcohort of 240 individuals with normal RBD score at baseline were included to develop the RBD prognostic model. One hundred and twenty-six subjects (52.5%) converted to RBD over the follow-up period while others remained negative. Cox proportional-hazards models demonstrated individuals with baseline abnormal UPSIT score, MDS-UPDRS total score, QUIP score and CSF t-tau levels had a higher risk of conversion from RBD-negativity to RBD-positivity (AUC 0.65 with 1 year; AUC 0.66 within 3 years; AUC 0.68 within 5 years; Figures 4D–F and Supplementary Table 4). Given the small sample size, the results of the RBD prognostic model should be interpreted with caution.

Furthermore, we explored the depression prognostic model in a subgroup of 322 subjects without depression at baseline. The predictive accuracy performed moderate using the combined variables (education years, MDS-UPDRS total score, MDS-UPDRS Part III score, UPSIT score, CSF Aβ_42_ and t-tau levels, Supplementary Table 4), and the ROC curves demonstrated an AUC of 0.79 within 1 year, 0.70 within 3 years and 0.70 within 5 years (Figures 4G–I).

### Internal validation and calibration of the prognostic models

Calibration plots of three prognostic models indicated a good agreement between predicted and observed probabilities (Supplementary Figure 2). Internal validation using the bootstrapping technique with 1,000 repetitions resulted in optimism corrected AUC within 5 years of 0.75 (cognitive decline model), 0.60 (RBD prognostic model), and 0.62 (depression prognostic model).

## Discussion

In the present study, we aimed to develop and validate clinical risk models using non-motor predictors to distinguish *de novo* PD from individuals. In addition, we constructed prognostic models for predicting the progression of non-motor symptoms (cognitive impairment, RBD, and depression) in *de novo* PD patients at 5 years of follow-up. The model validation established good discrimination and calibration for predicting PD onset and progression of non-motor symptoms.

Parkinson’s disease is a heterogeneous disorder, especially in the early disease course (Marras and Chaudhuri, 2016). Non-motor symptoms are prominent factors that influence fatality rate and mutilation rate in PD. It has long been recognized that many of them precede the motor features in many patients (Hely et al., 2005). In the models for distinguishing between early PD and HCs, we identified three general categories of predictors: clinical-related predictors, biomarker-related predictors and genetic-related predictors. Model 1 was developed with easily available and low-cost variables like demographics, health factors and functional assessments that can be widely used for screening PD risk in primary care settings. Model 2 added CSF and blood biomarkers to Model 1, and the final model (Model 3) reached a high accuracy with an AUC of 0.93 (95% CI: 0.91–0.96), which included three categories of predictors. The diagnostic performance in this study was similar to that of Nalls et al. (2015), with high accuracy and sensitivity (AUC 0.92, sensitivity 83.4%). Moreover, compared with their study, our analysis reported the CSF biomarkers’ influence on the disease risk models. Despite diagnostic decisions still relying on clinical features in practice, encouragingly, breakthroughs have been made recently in PD biomarker discovery (Parnetti et al., 2019). CSF biomarkers in PD (such as α-syn, Aβ_42_, tau, and NFL) have been suggested to possess the potential diagnostic and prognostic value of PD (Parnetti et al., 2019; Kwon et al., 2022). Tracking pathophysiological processes of PD, abnormal deposition of α-syn plays a critical role, which should become the foundation of composite biomarker panels (Majbour et al., 2016). In addition, biomarker-related factors, missense mutations as well as duplications in the α-syn protein-encoding *SNCA* gene are associated with *SNCA*-related parkinsonism, providing further support for a central neuropathological role of α-syn in PD (Kay et al., 2008; Rosborough et al., 2017). As the results showed, the value of logistic regression AUC improved slightly (0.02) after adding the CSF α-syn and *SNCA* rs356181 polymorphism, suggesting a predictive link of PD with α-syn levels. Approximately 10% of patients clinically diagnosed as PD have normal dopamine transporter (DAT) single-photon emission computed tomography (SPECT) imaging (Marek et al., 2014). This subgroup is referred to as having scans without evidence of dopaminergic deficit (SWEDD). In the present study, the exclusion of SWEDD participants from the PD model allows us to focus our efforts on more etiologically typical PD as defined by the clinical diagnosis and DAT scanning data. We also attempted an extended analysis to validate whether our diagnostic model could discriminate SWEDD from etiologically typical PD. The results suggested this model only achieved an AUC of 0.59 (95% CI: 0.52–0.66), with a low diagnostic value. Therefore, clinical features and non-motor symptoms cannot accurately distinguish between SWEDD and etiologically typical PD. DAT-SPECT imaging is a valuable diagnostic tool to help differentiate between PD and SWEDD, and imaging features will be taken into account to optimize our model in the future studies.

Longitudinal data provided the most substantial evidence on prognostic modeling, whereas relatively few previous studies accounted for longitudinal measurements when constructing NMS progression models. Our findings indicated that the AUC of prognostic models improved slightly with follow-up time. The overall AUCs fluctuated around 0.70. The present findings proved that participants with abnormal accumulation of amyloid, tau, older age at onset, higher level of LEDD, a lower level of education, abnormal measurements of UPSIT, MoCA, and RBDSQ had a significantly higher likelihood of cognitive decline. Similar results were suggested in a previous study which tested the five variables (age, UPSIT score, RBDSQ, CSF Aβ_42_, and mean caudate uptake) by logistic regression analysis and generated the AUC of 0.80 (95% CI: 0.74–0.87) (Schrag et al., 2017). Differently, our models possessed apparent advantages in predicting prognostic risk in multiple time dimensions. Besides, the early identification of patients at risk for depression and depression-related predictors as soon as possible is necessary to improve the quality of life (Reijnders et al., 2008). Previous studies also reported the association of several clinical information and CSF biomarkers with development of depression in PD using machine learning algorithm methods (Byeon, 2020a; Gu et al., 2020). Compared with our cohort, Gu et al. (2020) reported a slightly higher predictive value (AUC 0.94, 95% CI: 0.89–0.99). Similarly, the findings suggested that RBD and education levels were associated with depression in PD in previous studies, which supported our results (Byeon, 2020a). Sleep behavior disorders could serve as prodromal markers with a high risk for predicting neurodegeneration, and there has been a strong correlation with depression (Postuma et al., 2013). The presence of RBD in early PD patients may be a key determinant of increased risk of functional dependency, which indicated that RBD portended an unfavorable prognosis in Parkinson’s processes (Kim et al., 2019). We constructed the RBD prognostic model that could detect conversion from RBD-negativity to RBD-positivity with moderate accuracy. This finding was in line with the recent study, which developed a model for predicting the high-risk groups of RBD using random forest model with the prediction accuracy of 71.5% (Byeon, 2020b). Together, the UPSIT score was selected as a final predictor in three prognostic models. Our findings suggested that olfactory impairment may be a significant predictor predicting the occurrence of non-motor symptoms in PD, particularly cognitive decline. Considering the low cost and ease of assessment, olfactory impairment has become an attractive biomarker and also correlated with other non-motor features that may present later in the disease course (Fullard et al., 2017).

Our research possesses some strengths. Firstly, in our cohort, we constructed clinical risk models using non-motor predictors to distinguish between early PD and healthy individuals. In addition, we developed prognostic models for predicting the progression of non-motor symptoms among *de novo*, untreated PD. This study is the most comprehensive analysis of predictive models available in PD diagnosis and progression, keeping with overall assessments of the list of risks proposed in current clinical guidelines (Lennaerts et al., 2017). Additionally, this study provided relatively convenient methods, with low-cost and easily available clinical information as model features, which made the models feasible for practical application. Furthermore, in order to predict non-motor symptoms progression based on the patient’s baseline clinical data, clinicians can embed the predictive models in the electronic medical record system. The PPMI database collected data from multiple hospitals, which can improve the accuracy of our predictive models. The database describes a dynamic process of repeating measurements of clinical data annually with a higher degree of practical clinical application value.

There are also several potential limitations. First, the sample size is limited, especially a few participants were excluded from this study for missing records for CSF biomarkers and genetic assessments, which may cause bias in the final results. Future, more comprehensive research in larger cohorts is required to define prediction accuracy of models. Although the models were validated internally, developing risk model is still a work in progress that requires continuous refinement and revalidation in different cohorts. Besides, the data of this study were obtained from the PPMI database, which is not particularly appropriate to represent the general PD population, as it is an early study within 2 years of diagnosis. Further studies with more extended follow-up periods may enable long-term predictions.

## Conclusion

In total, the findings of our study facilitate predicting the individual risk at an early stage based on the predictors derived from these models. These predictive models provide reliable information to prevent PD onset and progression and further establish management strategies. Further research in large cohorts should explore how the clinical measurements and biomarkers combinations would present the best value for clinical and research purposes. Finally, future validation analysis is still needed to clarify these findings and provide more insight into the prognostic models over more extended periods of disease progression in more diverse samples.

## Data availability statement

The original contributions presented in this study are included in the article/Supplementary material, further inquiries can be directed to the corresponding author.

## Ethics statement

Written informed consent was obtained from the individual(s) for the publication of any potentially identifiable images or data included in this article.

## Author contributions

KD participated in the design of the study, drafted the manuscript, and carried out the conceptualization of the study. JM, XZ, WS, and MT performed the data analysis and drafted the manuscript. AX carried out the conceptualization of the study, reviewing, and critiquing the article at the same time. All authors contributed to the article and approved the submitted version.
