# Supplementary material for: Multi-predictor modeling for predicting early Parkinson’s disease and non-motor symptoms progression
Source: Front Aging Neurosci. 2022 Aug 26;14:977985. doi: 10.3389/fnagi.2022.977985 (PMC9459236; doi:10.3389/fnagi.2022.977985)
Supplement: Supplementary file 1 [file Data_Sheet_1.PDF]

Supplementary Material

Supplementary Table 1 Basic characteristics of study population in progression models

Characteristics	Cognition model	RBD model	Depression model
Age (mean, SD)	61.4 (9.9)	61.8 (9.6)	61.9 (9.7)
Gender (male/female)	200/109	150/90	216/106
Education years (mean, SD)	15.8 (2.8)	15.6 (3.0)	15.7 (2.9)
Famliy history of PD (Any Family with PD/No Family with PD)	74/234	57/183	82/240
Ethnicity (Hispanic or Latino/Not Hispanic or Latino)	6/303	5/235	6/316
Age of PD onset	59.4 (10.0)	59.7 (9.9)	59.9 (9.7)
Disease duration	6.5 (6.4)	6.7 (6.6)	6.8 (6.7)
MOCA score (mean, SD)	27.2 (2.3)	27.2 (2.3)	27. 1 (2.3)
UPSIT score (mean, SD)	22.7 (8.2)	22.9 (7.9)	22.3 (8.0)
RBDSQ score (No RBD/RBD)	200/109	240/0	210/112
GDS core (not depressed/depressed)	271/38	213/27	322/0
ESS score (not sleepy/sleepy)	269/40	213/27	282/40
MDS-UPDRS Part III score (mean, SD)	20.7 (8.7)	20.6 (8.7)	20.5 (8.9)
MDS-UPDRS total score (mean, SD)	31.9 (12.9)	30.4 (8.7)	30.9 (12.6)
Total Rigidity Score	3.9 (2.7)	3.7 (2.5)	3.7 (2.6)
TD/PIGD classification (non-TD or PIGD /TD)	93/216	69/171	88/234
Tremor score (OFF, mean, SD)	4.3 (3.2)	4.4 (3.4)	4.3 (3.2)
CSF α-synuclein (pg/ml, mean, SD)	1543.1 (684.7)	1526.4 (651.1)	1534.2 (648.50
CSF $A\beta_{42}$ (pg/ml, mean, SD)	948.9 (426.2)	911.5 (378.8)	940.8 (416.0)
CSF total tau (pg/ml, mean, SD)	170.1 (56.9)	165.2 (54.1)	179.0 (56.8)

PD, Parkinson's disease; SD, standard deviation; MOCA, Montreal Cognitive Assessment; UPSIT, University of Pennsylvania Smell Inventory Test; RBDSQ, Rapid-eye-movement sleep Behavior Disorder Screening Questionnaire; ESS, Epworth Sleeping Scale; GDS, Geriatric Depression Scale; MDS-UPDRS, Movement Disorder Society-Sponsored Revision of the Unified Parkinson's Disease Rating Scale; TD, Tremor Dominate; PIGD, Postural Instability/Gait Disorder; CSF, cerebrospinal fluid; $A\beta_{42}$, Amyloid-β_{42}.

Supplementary Table 2 Coefficients of each diagnostic model from backward stepwise logistic regressions

Variables	Model 1		Model 2		Model 3	
	Coefficients	P	Coefficients	P	Coefficients	P
Age	0.04	0.003	0.03	0.055	0.02	0.020
Gender	0.53	0.036	0.73	0.010	0.86	0.006
Famliy history of PD	1.64	9.21E-05	1.59	4.43E-04	1.66	0.001
UPSIT score	-0.26	<2E-16	-0.27	<2E-16	-0.30	<2E-16
MOCA score	-0.31	1.65E-04	-0.31	5.45E-04	-0.33	0.001
RBDSQ score	0.64	0.023	0.50	0.107	0.69	0.042
CSF α-synuclein			-0.53	0.131	-0.53	0.034
SNCA_rs356181					-0.61	0.005

PD, Parkinson's disease; MOCA, Montreal Cognitive Assessment; UPSIT, University of Pennsylvania Smell Inventory Test; RBDSQ, Rapid-eye-movement sleep Behavior Disorder Screening Questionnaire; CSF, cerebrospinal fluid.

Supplementary Table 3 The correlation coefficient values between the final variables in the diagnostic model (Model 3)

	Age	Gender	Family history	UPSIT	RBDSQ	MOCA	CSF α-syn	SNCA rs356181
Age	1	0.09	-0.07	-0.22	0.01	-0.184	0.143	0.05
Gender	0.09	1	0.05	-0.15	0.03	0.148	-0.073	-0.02
Family history	-0.07	0.05	1	-0.18	0.04	0.042	-0.039	0.03
UPSIT	-0.22	-0.15	-0.18	1	-0.18	0.228	0.068	0.06
RBDSQ	0.01	0.03	0.04	-0.18	1	0.026	-0.027	0.01
MOCA	-0.18	0.15	-0.04	0.23	-0.03	1	0.017	0.06
CSF α-syn	0.14	-0.07	-0.04	0.07	-0.03	0.0169	1	0.04
SNCA rs356181	0.05	0.02	0.03	0.06	0.01	0.061	0.044	1

UPSIT, University of Pennsylvania Smell Inventory Test; RBDSQ, Rapid-eye-movement sleep Behavior Disorder Screening Questionnaire; MOCA, Montreal Cognitive Assessment; CSF, cerebrospinal fluid; α-syn, α-synuclein.

Supplementary Table 4 Coefficients of progression models from cox risk regression

Variables	Coefficients	SE (coefficients)	z	P
Cognition model				
Age of PD onset	0.030	0.012	2.598	0.009
Education years	-0.065	0.036	-1.818	0.069
CSF Aβ_{42}	-0.001	3.09E-04	-4.474	7.68E-06
CSF total tau	0.004	0.002	2.273	0.023
UPSIT score	0.030	0.013	-2.277	0.023
MOCA score	-0.085	0.043	-1.974	0.023
RBDSQ score	0.350	0.201	1.737	0.050
LEDD	2.27E-04	8.018e-05	2.833	0.005
RBD model				
Age of PD onset	-0.019	0.011	-1.814	0.069
UPSIT score	-0.036	0.012	-2.852	0.004
QUIP score	0.428	0.220	1.941	0.052
CSF total tau	-0.003	0.002	-1.895	0.058
MDS-UPDRS total score	0.021	0.008	2.473	0.013
Depression model				
Education years	-0.077	0.041	-1.9	0.057
UPSIT score	-0.035	0.016	-2.245	0.025
CSF total tau	0.006	0.002	2.889	0.004
CSF Aβ_{42}	-7.10E-04	3.14E-04	-2.265	0.024
MDS-UPDRS total score	0.081	0.019	4.189	2.80E-05
MDS-UPDRS Part III score	-0.097	0.028	-3.412	6.44E-04

PD, Parkinson’s disease; MOCA, Montreal Cognitive Assessment; UPSIT, University of Pennsylvania Smell Inventory Test; RBDSQ, Rapid-eye-movement sleep Behavior Disorder Screening Questionnaire; MDS-UPDRS, Movement Disorder Society-Sponsored Revision of the Unified Parkinson’s Disease Rating Scale; CSF, cerebrospinal fluid; Aβ_{42}, Amyloid-β_{42}.

Supplementary Figure 1 Calibration plot for the diagnostic model (Model 3)

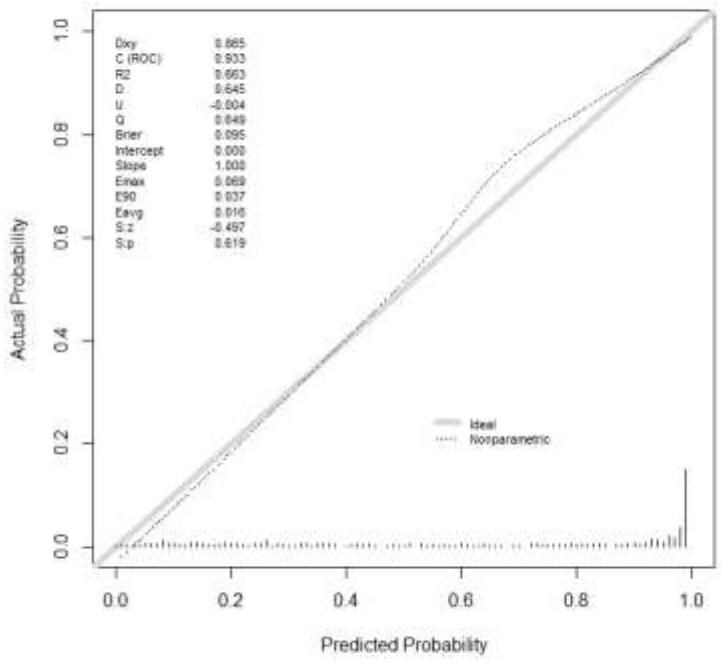

Diagonal line indicates perfect calibration.

Supplementary Figure 2 Calibration plots for predicting non-motor symptoms progression within 5 years

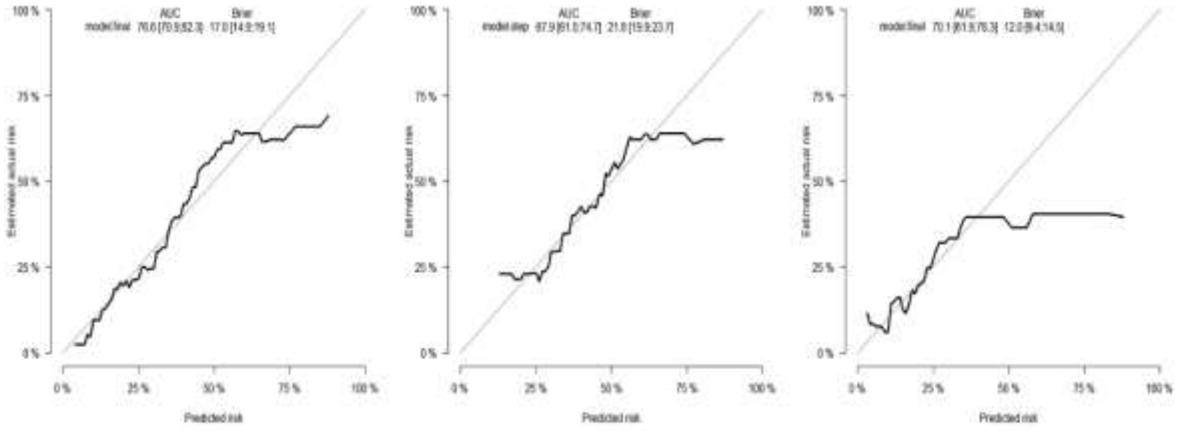

A B C

(A) cognitive decline model (B) RBD prognostic model (C) depression prognostic model
Diagonal line indicates perfect calibration.
